# Diploid genome assembly of *Kluyveromyces marxianus* NRRL Y-50883 (SLP1)

**DOI:** 10.1093/g3journal/jkab347

**Published:** 2021-10-13

**Authors:** Carolina Gómez-Márquez, Dania Sandoval-Nuñez, Anne Gschaedler, Teresa Romero-Gutiérrez, Lorena Amaya-Delgado, J Alejandro Morales

**Affiliations:** 1 Departamento de Ciencias Computacionales, Centro Universitario de Ciencias Exactas e Ingenierías, Universidad de Guadalajara, Guadalajara 44430, México; 2 Biotecnología Industrial, Centro de Investigación y Asistencia en Tecnología y Diseño del Estado de Jalisco A.C, Zapopan 45019, México

**Keywords:** *Kluyveromyces marxianus*, SLP1, *non-Saccharomyces yeast*, complete genome, gene annotation, nondairy yeast

## Abstract

The yeast *Kluyveromyces marxianus* SLP1 has the potential for application in biotechnological processes because it can metabolize several sugars and produce high-value metabolites. *K. marxianus* SLP1 is a thermotolerant yeast isolated from the mezcal process, and it is tolerant to several cell growth inhibitors such as saponins, furan aldehydes, weak acids, and phenolics compounds. The genomic differences between dairy and nondairy strains related to *K. marxianus* variability are a focus of research attention, particularly the pathways leading this species toward polyploidy. We report the diploid genome assembly of *K. marxianus* SLP1 nonlactide strain into 32 contigs to reach a size of ∼12 Mb (N50 = 1.3 Mb) and a ∼39% GC content. Genome size is consistent with the *k*-mer frequency results. Genome annotation by Funannotate estimated 5000 genes in haplotype A and 4910 in haplotype B. The enriched annotated genes by ontology show differences between alleles in biological processes and cellular component. The analysis of variants related to DMKU3 and between haplotypes shows changes in *LAC12* and *INU1*, which we hypothesize can impact carbon source performance. This report presents the first polyploid *K. marxianus* strain recovered from nonlactic fermenting medium.

## Introduction 

The yeast *Kluyveromyces marxianus* is emerging as a remarkable alternative for fermentation processes, metabolic engineering, omics analysis, and recombinant protein production, having several advantages over other yeasts. These include the ability to metabolize a wide variety of sugars, such as glucose, fructose, fructans, xylose, and lactose, which are abundant sugars in nature (*i.e.*, agave juice, lignocellulosic biomass, whey, and molasses) (Fonseca *et al.* 2008; Lane and Morrissey 2010; Amaya-Delgado *et al.* 2013; Flores *et al.* 2013; Schabort *et al.* 2018).

*K. marxianus* is thermotolerant and shows rapid cellular growth even when cultured with growth-inhibitor molecules (Li *et al.* 2018; Flores-Cosío *et al.* 2019). Previous studies of this yeast highlighted the significant physiological variation amongst *K. marxianus* strains in terms of their fermentation characteristics and ability to use various substrates, suggesting genetic diversity within the species (Flores *et al.* 2013; Ortiz-Merino *et al.* 2018).

Our strain, *K. marxianus* NRRL Y-50883 (SLP1), was isolated from hostile fermentation musts during the mezcal fermentation process in San Luis Potosí, México, and belongs to the yeast collection of the Centro de Investigación y Asistencia en Tecnología y Diseño del Estado de Jalisco (CIATEJ, A.C). *K. marxianus* SLP1 is a strain with interesting characteristics because it produces ethanol from mixtures of glucose–xylose, and agave fructans and fructose, while producing several industry-desirable metabolites such as fructanases, aromatic compounds, and higher alcohols (Flores *et al.* 2013; Flores-Cosío et al. 2018).

Compared to other yeasts, SLP1 produces a significant amount of ethanol, derived from different lignocellulosic hydrolysates and agave juice for tequila production (Flores *et al.* 2013; Sandoval-Nuñez *et al.* 2018; Alcazar-Valle *et al.* 2019). SLP1 can tolerate high concentrations of organic acids, furans, phenols, oxidant agents, and saponins, which are considered growth inhibitors, as well as high temperatures. This outstanding tolerance is related to the physiological ability of SLP1 to change the cell membrane composition to increase its robustness under unfavorable fermentation conditions (Arellano-Plaza *et al.* 2013; Alcázar *et al.* 2017; Mejía-Barajas *et al.* 2017, 2018; Flores-Cosío *et al.* 2019).

To understand the genomic differences of *K. marxianus* SLP1 and other strains of this species that can be isolated, for example, from dairy products, it is important to know the genetic features that provide thermotolerance, sugar transporter, and other traits that confer the most significant qualities of SLP1. Ortiz-Merino *et al.* (2018) showed divergence in *K. marxianus* UFS-Y2791 isolated from *Agave Americana* compared to *K. marxianus* isolated from dairy products. This opens the question as to what extent *K. marxianus* genomic variability is associated with nondairy strains.

Here, we report the whole-genome sequencing, assembly, and annotation results of *K. marxianus* SLP1, which allow the identification of the genomic machinery available in this strain as a product of the adaptive response to a stress phenomenon and the evaluation of its impact on this mechanism through gene identification, enrichment by ontology, and variant classification. The results of this work allow us to study the dynamics of adaptation and provide knowledge on nonlactic *K. marxianus* that show diploidy.

## Materials and methods

### Yeast strain and library preparation

The yeast *K.* *marxianu*s (NRRL Y-50883) was grown overnight at 30°C at 250 rpm in 50 mL of YPD medium to the early stationary phase before cells were harvested by centrifugation. Total genomic DNA was extracted using the Promega Wizard^®^ Genomic DNA Purification Kit Technical Manual according to the manufacturer’s instructions. DNA quality was verified using a Nanodrop ND-1000 spectrophotometer (Thermo Scientific, Wilmington, Delaware, USA) and verifying its integrity in agarose gel.

### PacBio sequencing

The sequencing was performed by Macrogen Inc, Korea. Whole-genome *de novo* sequencing was performed by PacBio using a SMRT Cell 8Pac V3 with DNA Polymerase Binding Kit P6 and DNA Sequencing Reagent 4.0 v2. The reads were processed by the parallel statistical analysis of the k-mer spectrum in sequencing Jellyfish data (Marçais and Kingsford 2011), which counts the occurrence of all 21-mer length fragments. Once the raw data were processed, they were moved to GenomeScope 2.0, a reference-free analyzer used to establish genome profiling (Ranallo-Benavidez *et al.* 2020). PacBio performed exploration with both tools, and generated outputs estimates for size, repetitiveness, and polyploid (through heterozygosity rate).

### Genome assembly

*K. marxianus* SLP1 raw reads were assembled using Falcon software via Bioconda (https://github.com/PacificBiosciences/pb-assembly). The first Falcon module was run in three steps: (1) overlap detection and error correction of raw reads, (2) overlap detection of corrected reads, and (3) string graph assembly. Following, to extract haplotig fragments, we ran the Falcon-Unzip package with the previously generated data. The two main stages for the latter were: (1) identifying SNPs and assigning phases and (2) assembling the graph annotation with the phases. Falcon-Unzip was run with the parameters adjusted for yeast sequences. After, we generated two types of fasta files: primary contigs and haplotigs. Primary contigs are the longest continuous stretches of contiguously assembled sequences, and haplotigs are contigs from a specific haplotype (Chin *et al.* 2016).

### Quality assembly

The assembly quality was assessed using the QUAST 5.1 (https://github.com/ablab/quast) algorithm. We calculated basic metrics (L50, N50, and number of sequences, amongst others) and included NG50 and L50 using a reference genome *K. marxianus* DMKU3 (*K.* *marxianus* GCA_001417885.1). The genome completeness was determined using Benchmarking Universal Single-Copy Orthologs (BUSCO) software v4.1.4 with the Saccharomycetes ortholog gene set from OrthoDB in terms of found single-copy orthologs in the genome by researching the absence or presence of highly conserved genes, which are reported as complete if the length is two standard deviations of BUSCO groups, duplicated if a complete gene is found with more than one copy, fragmented if is partially recovered, and missing if is not recovered (Simão *et al.* 2015).

### Variant identification and quality control

We also aligned the contigs with the reference genome to find structural variants with the MUMmer package nucmer. Sequences were aligned with MUMmer 3.23 using a reference genome (*K. marxianus* DMKU3-1043 with genome assembly identifier: GCA_001417885.1), and by calling the SNP positions, the output file was converted to a VCF file. Next, variant annotation was analyzed by SNPeff 5.0 using the public DMKU3-1042 genome annotation as a reference. In addition, we aligned the amino acid sequences of the SLP1 alleles to two genes using the InterProScan web service (https://www.ebi.ac.uk/interpro/search/sequence/): *LAC12* with strain CBS 397 for its efficient lactose consumption, and *INU1* with strain DMKU3 for sequence similarity.

### Genome annotation

We separated the *K. marxianus* SLP1 diploid genome into haplotypes to identify both sub-genomes. After the assembly, we assigned primary contigs as haplotype A. Then, we designed a Python (3.8) script that takes the coordinate data of each haplotig produced by Falcon, replacing the sequences in the primary contig to generate the haplotype B.

Gene prediction and annotation were conducted in both haplotypes with Funannotate pipeline v. 1.7.0 (Palmer 2016), which includes softmasking with RepeatMasking and RepeatModeler tools, an *ab initio* gene predictor (Augustus, snap, glimmerHMM, codingQuary, and GenMark), and functional annotation of the protein-coding genes. Then, we assessed the ontology enrichment of genes categorized by the PANTHER web server (http://pantherdb.org/) into the three main ontologies: molecular function, biological process, and cellular component. An NJ phylogenetic tree was generated with CLC Sequence Viewer 8.0 to establish taxonomic distance using the *K. marxianus* SLP1 fatty acid synthase subunit beta (*FAS1*) gene. The *FAS1* gene is present during different stress responses, considered a wide-range fermentative yeasts taxonomic marker (Roscini *et al.* 2020) and was proven to be essential in the adaptive evolution of *K. marxianus*.

## Results and discussion

### Sequencing and size estimation

Sequencing was performed on the PacBio RS System using Single Molecule Real Time (SMRT), generating 82,360 reads with an average length 10,384 bp for a total length of >855 Mb with N50 = 16,868 b.

**Figure 1:**
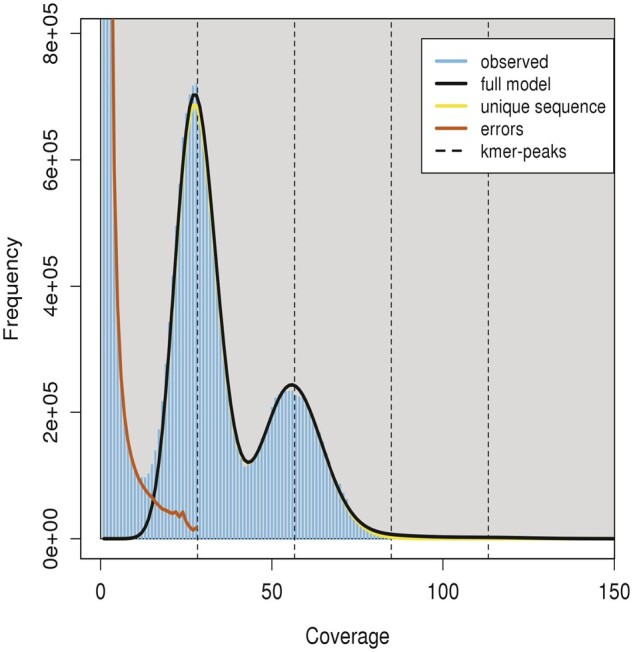
k-mer frequency of PacBio raw reads. Plot obtained by GenomeScope using *k* = 21. The fit of the GenomeScope model (black line) and the k-mer frequencies observed (blue area).

Pacbio reads showed a high heterozygosity rate, consistent with our previous analysis of *K. marxinus* SLP1 k-mer frequency in sequencing data produced by the Illumina platform (data not shown). Figure  1 shows the k-mer profile results from GenomeScope. There are two major peaks between ∼30 and 55. The first peak is higher than the second; this is an indicator of high heterozygosity. This evidence is consistent with the findings of a diploid genome (Ranallo-Benavidez *et al.* 2020). The size estimation obtained by GenomeScope is 11.9 Mb, with a uniqueness of 88.33% and heterozygosity of 3.27%. After k-mer profile analysis, 70× coverage of the estimated 11.9 Mb *K. marxianus* SLP1 genome was calculated.

### *Kluyveromyces marxianus* assembly and quality measures

The *K. marxianus* SLP1 diploid genome was assembled into 32 primary contigs covering 12.04 Mb with N50 = 1.3 Mb and L50 = 4, with the same values for NG50 and LG50. We also identified 38 haplotigs covering 955,365bp with N50 = 368,823bp, NG50 = 361, 944 bp, and L50 = 9, with the same value for LG50, which means that our contigs are same length and quantity in terms of genome average in comparison to *K. marxianus* DMKU3. The smallest fragment in the primary contigs is 20,533 and 3040 bp in haplotigs as we show in Table  1.

**Table 1:** *K. marxianus* SLP1 summary assembly. Statistics of primary contigs and haplotigs. NG50 and LG50 were calculated using *K. marxianus* DMKU3 as the reference.

	Primary contigs	Haplotigs
Sequences	32	38
GC %	39.91	39.13
N50	1,343,707 bp	368,823 bp
NG50	1,343,707 bp	361,944 bp
L50	4	9
LG50	4	9
Assembly length	12,044,577 bp	955,365 bp

**Figure 2:**
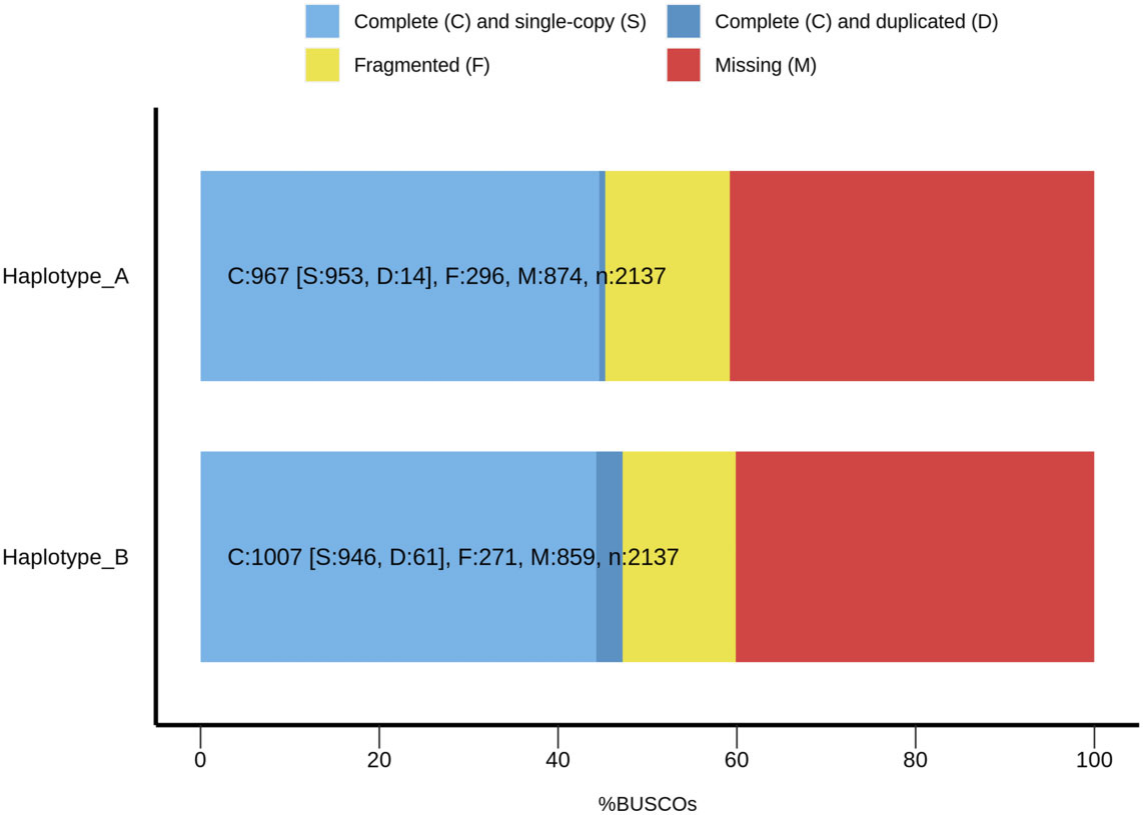
BUSCO summary of *K. marxianus* SLP1 haplotypes. Haplotype A and haplotype B of *K. marxianus* SLP1 with Saccharomycetes orthology data from orthoDB represented by the number of universal single-copy reference orthologues (BUSCOs). The blue and dark blue areas represent the number of complete and single-copy genes, and complete and duplicated genes, respectively; yellow and red indicate the number of fragmented and missing genes, respectively; and *n* is the number of genes used.

The completeness of the genome as determined by BUSCO software is shown in Figure  2, where haplotypes A and B have 14 and 61 genes, respectively, appearing as duplicated copies due to the divergence between haplotypes. Conversely, 60% of the genes are complete and fragmented, while 40% are missing. Missing genes may be due to either no significant matches, or the BUSCO matches scored below the range of scores for the BUSCO profile. The missing genes could also be due to the assembler used (Falcon) and its limitation with mitochondrial sequences as reported by Hamlin *et al.* (2019). The assembly completeness in the gene contend term can be used to measure the quality of the assembly, but it is for known genes that are incorporated in databases, and high-quality genomes may reflect the organism’s evolutionary history rather than an incomplete assembly.

### *Kluyveromyces marxianus* annotation

Funannotate pipeline reported 4732 predicted CDS in haplotype A and 4646 in haplotype B. The summary annotation is shown in Table  2, which lists the number of genes, CDS, and tRNAs. Each haplotype was annotated separately; the results show 1429 gene IDs in haplotype A and 1341 gene IDs in haplotype B. The remaining CDS are reported as hypothetical proteins, so we decided to work only with CDS with known function.

**Table 2:** *K. marxianus* SLP1 haplotype annotation summary

	Haplotype	
	A	B
Genes	5000	4910
CDS	4732	4646
tRNA	268	264

**Figure 3:**
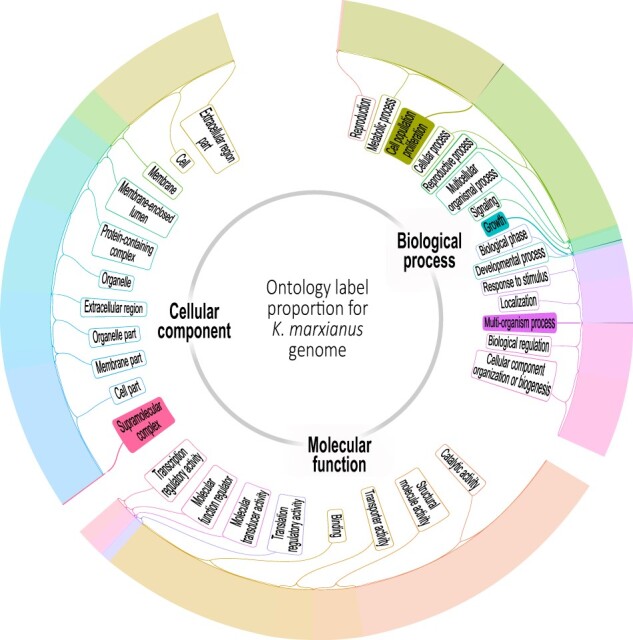
Ontology enrichment. The bar graphs surrounding the plot circumference are represented by the relative frequency of genes in each ontology term. Nonfilled boxes are ontologies present in both haplotypes while color-filled boxes represent functions present in only one haplotype.

The enrichment by ontologies in annotated genes in Table  2 is presented by genes in each haplotype, as we described in the Methods section. Figure  3 shows the ontologies present in our yeast strain where some functions are different between haplotypes. Such gained functions could be the consequence of the characteristic variability generated during sexual reproduction that occurs -in the case of *K. marxianus*- under stressful conditions. The genome contains 187,219 SNPs, 36,674 INS, and 70,565 DEL in haplotype A, and 210,342 SNPs, 41,416 INS, and 68,323 DEL in haplotype B, as reported by Snpeff. In addition, we report the effect of variants at the *INU1* gene because its influence on fructan assimilation is a trait of this strain. Table  3 shows 198 variants located in the up- and down-stream regions have an impact as modifiers. However, there are variants have high, low, and moderate impacts. Those with high impact are frameshift variants caused by INDEL, those with moderate impact are missense variants, and those with low impact are synonymous variants. The phylogenetic tree (Supplementary Figure S1) shows the distances between *Saccharomyces cerevisiae*, *K. marxianus*, *Kluyveromyces lactis*, and other related species.

**Table 3:** Variant annotation summary. High, frameshift variant; low, synonymous variant; moderate, missense variant; modifier, up- and downstream gene variant.

Chromosome	Gene	Coordinate	High	Low	Moderate	Modifier
1	INU1	1,092,964 to 1,098,595	11	5	1	198

## Relevance

**Figure 4:**
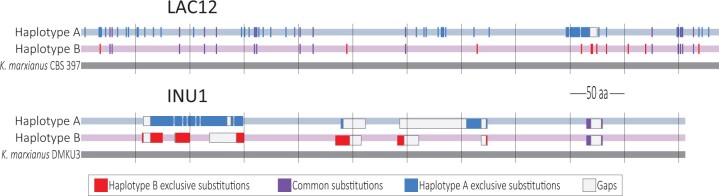
Alignment scheme of *LAC12* and *INU1* genes. LAC12 amino acid sequence alignment representation from *K. marxianus* CBS 397 to SLP1 haplotypes. *INU1* amino acid sequence alignment representation from *K. marxianus* DMKU3 to SLP1 haplotypes. Common substitutions are represented by purple lines, red lines indicate haplotype B exclusive substitutions, and blue lines indicate haplotype A exclusive substitutions. Gaps in the alignment are indicated by white boxes.

This is the first report of a diploid *K. marxianus* living in a fermenting agave must medium. All other polyploid *K. marxianus* have been isolated from lactic mediums, and Ortiz-Merino *et al.* (2018) demonstrated that *LAC12* (functional and nonfunctional) gene alleles are associated with their chromosome set number. Mo *et al.* (2019) showed that *K. marxianus* FIM1, a haploid yeast isolated from nonlactic environments, was highly stable when grown in ethanol-rich medium; our report underlies the need to further study the mechanisms of adaptive polyploidy evolution in fructan-rich growing-medium *K. marxianus*. Such evidence opens the question if using other carbon-sugar sources is related to the ploidy phenomenon. The ability of *K. marxianus* SLP1 to consume a variety of substrates and to cope with hostile environments suggests a sexual recombination event that allowed functional gene gain in this strain. Figure  4 depicts the alignment representation of the LAC12 gene from *K. marxianus* CBS 397 to *K. marxianus* SLP1 haplotypes. We observe that the sequence of the haplotype A contains the largest number of exclusive substitutions, whereas haplotype B shows the highest homology with the *LAC12* reference gene. However, we observe several exclusive substitutions and gaps in both haplotypes compared to the *INU1* gene from *K. marxianus* DMKU3.

## Data availability

Raw reads are available in the National Center for Biotechnology Information (NCBI) with SRA accession number SRR14000474; the genome assembly generated in this study was deposited under BioProject ID PRJNA714250.

Supplementary material is available at *G3* online.

## Funding

Financial support is kindly acknowledged from the Energy Sustainability Fund 2014 (CONACYT-SENER, Mexico) Grants 245750. Sandoval-Nuñez D. and Gómez-Márquez C. acknowledge financial support from CONACYT in the form of Ph.D. scholarships 487000 and 443922, respectively.

## Conflicts of interest

The authors declare that there is no conflict of interest.

## Supplementary Material

jkab347_Supplementary_Data

jkab347_Supplementary_Figure
